# Effects of eight weeks of time-restricted feeding (16/8) on basal metabolism, maximal strength, body composition, inflammation, and cardiovascular risk factors in resistance-trained males

**DOI:** 10.1186/s12967-016-1044-0

**Published:** 2016-10-13

**Authors:** Tatiana Moro, Grant Tinsley, Antonino Bianco, Giuseppe Marcolin, Quirico Francesco Pacelli, Giuseppe Battaglia, Antonio Palma, Paulo Gentil, Marco Neri, Antonio Paoli

**Affiliations:** 1 Department of Biomedical Sciences, University of Padova, Padua, Italy; 2 Department of Kinesiology & Sport Management, Texas Tech University, Lubbock, TX USA; 3 Sport and Exercise Sciences Research Unit, University of Palermo, Palermo, Italy; 4 Italian Fitness Federation, Ravenna, Italy; 5 College of Physical Education and Dance, Federal University of Goias, Goiania, Brazil

**Keywords:** Intermittent fasting, Time-restricted feeding, Resistance training, Body composition, Body builders, Fasting

## Abstract

**Background:**

Intermittent fasting (IF) is an increasingly popular dietary approach used for weight loss and overall health. While there is an increasing body of evidence demonstrating beneficial effects of IF on blood lipids and other health outcomes in the overweight and obese, limited data are available about the effect of IF in athletes. Thus, the present study sought to investigate the effects of a modified IF protocol (i.e. time-restricted feeding) during resistance training in healthy resistance-trained males.

**Methods:**

Thirty-four resistance-trained males were randomly assigned to time-restricted feeding (TRF) or normal diet group (ND). TRF subjects consumed 100 % of their energy needs in an 8-h period of time each day, with their caloric intake divided into three meals consumed at 1 p.m., 4 p.m., and 8 p.m. The remaining 16 h per 24-h period made up the fasting period. Subjects in the ND group consumed 100 % of their energy needs divided into three meals consumed at 8 a.m., 1 p.m., and 8 p.m. Groups were matched for kilocalories consumed and macronutrient distribution (TRF 2826 ± 412.3 kcal/day, carbohydrates 53.2 ± 1.4 %, fat 24.7 ± 3.1 %, protein 22.1 ± 2.6 %, ND 3007 ± 444.7 kcal/day, carbohydrates 54.7 ± 2.2 %, fat 23.9 ± 3.5 %, protein 21.4 ± 1.8). Subjects were tested before and after 8 weeks of the assigned diet and standardized resistance training program. Fat mass and fat-free mass were assessed by dual-energy x-ray absorptiometry and muscle area of the thigh and arm were measured using an anthropometric system. Total and free testosterone, insulin-like growth factor 1, blood glucose, insulin, adiponectin, leptin, triiodothyronine, thyroid stimulating hormone, interleukin-6, interleukin-1β, tumor necrosis factor α, total cholesterol, high-density lipoprotein cholesterol, low-density lipoprotein cholesterol, and triglycerides were measured. Bench press and leg press maximal strength, resting energy expenditure, and respiratory ratio were also tested.

**Results:**

After 8 weeks, the 2 Way ANOVA (Time * Diet interaction) showed a decrease in fat mass in TRF compared to ND (p = 0.0448), while fat-free mass, muscle area of the arm and thigh, and maximal strength were maintained in both groups. Testosterone and insulin-like growth factor 1 decreased significantly in TRF, with no changes in ND (p = 0.0476; p = 0.0397). Adiponectin increased (p = 0.0000) in TRF while total leptin decreased (p = 0.0001), although not when adjusted for fat mass. Triiodothyronine decreased in TRF, but no significant changes were detected in thyroid-stimulating hormone, total cholesterol, high-density lipoprotein, low-density lipoprotein, or triglycerides. Resting energy expenditure was unchanged, but a significant decrease in respiratory ratio was observed in the TRF group.

**Conclusions:**

Our results suggest that an intermittent fasting program in which all calories are consumed in an 8-h window each day, in conjunction with resistance training, could improve some health-related biomarkers, decrease fat mass, and maintain muscle mass in resistance-trained males.

## Background

Fasting, the voluntary abstinence from food intake for a specified period of time, is a well-known practice associated with many religious and spiritual traditions. In fact, this ascetic practice is referenced in the Old Testament, as well as other ancient texts such the Koran and the Mahabharata. In humans, fasting is achieved by ingesting little to no food or caloric beverages for periods that typically range from 12 h to 3 weeks. Muslims, for example, fast from dawn until dusk during the month of Ramadan, while Christians, Jews, Buddhists, and Hindus traditionally fast on designated days or periods [1]. Fasting is distinct from caloric restriction (CR), in which daily caloric intake is chronically reduced by up to 40 %, but meal frequency is maintained [2]. In contrast to fasting, starvation is a chronic nutritional deficiency that is commonly incorrectly used as a substitute for the term “fasting”. Starvation could also refer to some extreme forms of fasting, which can result in an impaired metabolic state and death. However, starvation typically implies chronic involuntary abstinence of food, which can lead to nutrient deficiencies and health impairment. While a prolonged period of fasting is difficult to perform for the normal population, an intermittent fasting (IF) protocol has been shown to produce higher compliance [3]. Typically, IF is defined by a complete or partial restriction in energy intake (between 50 and 100 % restriction of total daily energy intake) on 1–3 days per week or a complete restriction in energy intake for a defined period during the day that extends the overnight fast. The most studied of the above form of IF is Ramadan fasting: during the holy month of Ramadan, which varies according to the lunar calendar, Muslims abstain from eating or drinking from sunrise to sunset. The effects of Ramadan have been extensively investigated, not only on health outcomes [1, 4–8], but also on exercise performance [9–16]. Moreover, in recent years a focus on other forms of IF, unrelated to religious practice, has emerged. One such form, alternate day fasting (ADF; fasting every other day) is organized with alternating “feast days,” on which there is an “ad libitum” energy intake, and “fast days” with reduced or null energy intake.

A growing body of evidence suggests that, in general, IF could represent an useful tool for improving health in general population due to reports of improving blood lipids [17–20] and glycaemic control [3], reducing circulating insulin [21], decreasing blood pressure [1, 21–23], decreasing inflammatory markers [7] and reducing fat mass even during relatively short durations (8–12 weeks) [23]. These reported effects are probably mediated through changes in metabolic pathways and cellular processes such as stress resistance [24], lipolysis [3, 17, 25–27], and autophagy [28, 29]. One particular form of IF which has gained great popularity through mainstream media is the so-called time-restricted feeding (TRF). TRF allows subjects to consume ad libitum energy intake within a defined window of time (from 3–4 h to 10–12 h), which means a fasting window of 12–21 h per day is employed. A key point concerning the IF approach is that generally calorie intake is not controlled, but the feeding times are.

In sports, IF is studied mainly in relationship with Ramadan period [9–16], whilst TRF has become very popular among fitness practitioners claiming supposed effects on maintenance of muscle mass and fat loss. Very limited scientific information is available about TRF and athletes, and mixed results have been reported [22, 30, 31]. We demonstrated very recently [30] that TRF did not affect total body composition nor had negative effects on muscle cross-sectional area after 8 weeks in young previously-untrained men performing resistance training, despite a reported reduction in energy intake of ~650 kcal per fasting day in the TRF group. Thus the aim of the present study was to investigate the effects of an isoenergetic TRF protocol on body composition, athletic performance, and metabolic factors during resistance training in healthy resistance trained males. We hypothesized that the TRF protocol would lead to greater fat loss and improvements in health-related biomarkers as compared to a typical eating schedule.

## Methods

### Subjects

Thirty-four resistance-trained males were enrolled through advertisements placed in Veneto region’s gyms. The criteria for entering the study were that subjects must have performed resistance training continuously for at least 5 years (training 3–5 days/week with at least 3 years experience in split training routines), be presently engaged in regular resistance training at the time of recruitment, be life-long steroid free, and have no clinical problems that could be aggravated by the study procedures.

Fifty-three subjects responded to the advertisement, but 7 were excluded for previous use of anabolic steroids, and 12 declined participation after explanation of study’s protocol. Therefore, 34 subjects (age 29.21 ± 3.8; weight 84.6 ± 6.2 kg) were randomly assigned to a time-restricted feeding group (TRF; n = 17) or standard diet group (ND; n = 17) through computer-generated software. The research staff conducting outcome assessments was unaware of the assignment of the subjects (i.e. a single blind design). Anthropometric baseline characteristics of subjects are shown in Table 1. All participants read and signed an informed consent document with the description of the testing procedures approved by the ethical committee of the Department of Biomedical Sciences, University of Padova, and conformed to standards for the use of human subjects in research as outlined in the current Declaration of Helsinki.

**Table 1:** Subject characteristics at baseline

	TRF	ND
Age	29.94 ± 4.07	28.47 ± 3.48
Weight (kg)	83.9 ± 12.8	85.3 ± 13
Height (cm)	178 ± 5	177 ± 4
FM (kg)	10.9 ± 3.5	11.3 ± 4.5
FFM (kg)	73.1 ± 5.7	73.9 ± 3.9

Results presented as mean ± SD. Results are not statistically significantly different

### Diet

Dietary intake was measured by a validated 7-day food diary [32–34], which has been used in previous studies with athletes [35], and analysed by nutritional software (Dietnext®, Caldogno, Vicenza, Italy). Subjects were instructed to maintain their habitual caloric intake, as measured during the preliminary week of the study (Table 2). During the 8-week experimental period, TRF subjects consumed 100 % of their energy needs divided into three meals consumed at 1 p.m., 4 p.m. and 8 p.m., and fasted for the remaining 16 h per 24-h period. ND group ingested their caloric intake as three meals consumed at 8 a.m., 1 p.m. and 8 p.m. This meal timing was chosen to create a balanced distribution of the three meals during the feeding period in the TRF protocol, while the schedule for the ND group maintained a normal meal distribution (breakfast in the morning, lunch at 1 p.m. and dinner at 8 p.m.). The distribution of calories was 40, 25, and 35 % at 1 p.m., 4 p.m. and 8 p.m. respectively for TRF, while ND subjects consumed 25, 40 and 35 % of daily calories at 8 a.m., 1 p.m. and 8 p.m. respectively. The specific calorie distribution was assigned by a nutritionist and was based on the reported daily intake of each subject.

**Table 2:** Diet composition and macronutrients distribution at basal level and during the experimental period in both groups

	TRF basal	TRF exp	ND basal	ND exp
Total (kcal/day)	2826 ± 412.3	2735 ± 386	3007 ± 444.7	2910 ± 376.4
Carbohydrates (kcal/day)	1503.4 ± 225.95	1400.3 ± 118.8	1654 ± 222.4	1609.2 ± 201.5
Fat (kcal/day)	698 ± 178.5	683.8 ± 61.6	728.7 ± 195	647.7 ± 183.4
Protein (kcal/day)	624.5. ± 59.5	650.3. ± 62.5	637 ± 72.9	643.1 ± 69.3
% Carbohydrates	53.2 ± 1.4	51.2 ± 3.6	54.7 ± 2.2	55.3 ± 4.2
% Fat	24.7 ± 3.1	25 ± 2.8	23.9 ± 3.5	22.6 ± 3.2
% Protein	22.1 ± 2.6	23.8 ± 3.1	21.4 ± 1.8	22.1 ± 3.2
Protein (g/kg_bw_)	1.86 ± 0.2	1.93 ± 0.3	1.9 ± 0.3	1.89 ± 0.4

Results presented as mean ± SD. No significant differences were detected between groups and within groups

ND subjects were instructed to consume the entire breakfast meal between 8 a.m. and 9 a.m., the entire lunch meal between 1 p.m. and 2 p.m., and the entire dinner meal between 8 p.m. and 9 p.m. TRF subjects were instructed to consume the first meal between 1 p.m. and 2 p.m., the second meal between 4 p.m. and 5 p.m., and the third meal between 8 p.m. and 9 p.m. No snacks between the meals were allowed except 20 g of whey proteins 30 min after each training session. Every week, subjects were contacted by a dietician in order to check the adherence to the diet protocol. The dietician performed a structured interview about meal timing and composition to obtain this information.

### Training

Training was standardized for both groups, and all subjects had at least 5 years of continuous resistance training experience prior to the study. Training consisted of 3 weekly sessions performed on non-consecutive days for 8 weeks. All participants started the experimental procedures in the months of January or February 2014.

The resistance training program consisted of 3 different weekly sessions (i.e. a split routine): session A (bench press, incline dumbell fly, biceps curl), session B (military press, leg press, leg extension, leg curl), and session C (wide grip lat pulldown, reverse grip lat pulldown and tricep pressdown). The training protocol involved 3 sets of 6–8 repetitions at 85–90 % 1-RM, and repetitions were performed to failure (i.e. the inability to perform another repetition with correct execution) with 180 s of rest between sets and exercises [36]. The technique of training to muscular failure was chosen because it is one of the most common practices for body builders, and it was a familiar technique for the subjects. As expected, the muscle action velocity varied between subjects due to their different anatomical leverage. Although there was slight variation of repetition cadence for each subject, the average duration of each repetition was approximately 1.0 s for the concentric phase and 2.0 s for the eccentric phase [37].

The research team directly supervised all routines to ensure proper performance of the routine. Each week, loads were adjusted to maintain the target repetition range with an effective load. Training sessions were performed between 4:00 and 6:00 p.m. Subjects were not allowed to perform other exercises other than those included in the experimental protocol.

### Measurements

Body weight was measured to the nearest 0.1 kg using an electronic scale (Tanita BWB-800 Medical Scales, USA), and height to the nearest 1 cm using a wall-mounted Harpenden portable stadiometer (Holtain Ltd, UK). Body mass index (BMI) was calculated in kg/m^2^. Fat mass and fat-free mass were assessed by dual energy X-ray absorptiometry (DXA) (QDR 4500 W, Hologic Inc., Arlington, MA, USA). Muscle areas were calculated using the following anthropometric system. We measured limb circumferences to the nearest 0.001 m using an anthropometric tape at the mid-arm and mid-thigh. We also measured biceps, triceps, and thigh skinfolds to the nearest 1 mm using a Holtain caliper (Holtain Ltd, UK). All measurements were taken by the same operator (AP) before and during the study according to standard procedures [38, 39]. Muscle areas were then calculated using a previously [40] validated software (Fitnext®, Caldogno, Vicenza, Italy). Cross-sectional area (CSA) measured with Fitnext® has an r^2^ = 0.88 compared to magnetic resonance and an ICC of 0.988 and 0.968 for thigh and arm, respectively [40–42].

Ventilatory measurements were made by standard open-circuit calorimetry (max Encore 29 System, Vmax, Viasys Healthcare, Inc., Yorba Linda, CA, USA) with breath-by-breath modality. The gas analysis system was used: Oxygen uptake and carbon dioxide output values were measured and used to calculate resting energy expenditure (REE) and respiratory ratio (RR) using the modified Weir equation [43]. Before each measurement, the calorimeter was warmed according to the manufacturer’s instructions and calibrated with reference gases of known composition prior to each participant.

Oxygen uptake was measured (mL/min) and also normalized to body weight (mL/kg/min), and the respiratory ratio was determined. After resting for 15 min, the data were collected for 30 min, and only the last 20 min were used to calculate the respiratory gas parameters [37, 44]. All tests were performed in the morning between 6 and 8 a.m. while the subjects were supine. The room was dimly lit, quiet, and approximately 23 °C. Subjects were asked to abstain from caffeine, alcohol consumption and from vigorous physical activity for 24 h prior to the measurement.

### Blood collection and analysis protocol

Blood samples taken from the antecubital vein at baseline and after 8 weeks were collected in BD Vacutainers Tubes (SST™ II Advance, REF 367953). Samples were centrifuged (4000 RPM at 4 °C using centrifuge J6-MC by Beckman), and the resultant serum was aliquoted and stored at −80 °C. All samples were analysed in the same analytical session for each test using the same reagent lot. Before the analytical session, the serum samples were thawed overnight at 4 °C and then mixed. Interleukin-6 (IL-6), tumor necrosis factor-α (TNF-α), and interleukin-1β (IL-1β) were measured using Quantikine HS Immunoassay Kit (R&D Systems, Minneapolis, MN, USA). The inter-assay coefficient of variations (CVs) were 3.5–6.2 and 3.2–6.3 % for IL-6, TNF-α and IL-1β respectively. Insulin-like growth factor 1 (IGF-1) was measured using the analyzer Liaison XL (DiaSorin S.p.A, Vercelli-Italy). This test is a sandwich immunoassay based on a chemiluminescent revelation, and the CV for IGF-1 was between 5.6 and 9.6 %; the reference range for this test depends on age and gender. Fasting total cholesterol, high-density lipoprotein cholesterol (HDL-C), low-density lipoprotein cholesterol (LDL-C), and triglycerides (TG) were measured by an enzymatic colorimetric method using a Modular D2400 (Roche Diagnostics, Basel, Switzerland). LDL-C fraction was calculated from Friedewald’s formula: LDL-C = TC − HDL-C − (TG/5). The inter-assay CVs for total cholesterol, HDL-C, and triacylglycerol concentrations were 2.9, 1.8, and 2.4 %, respectively. Glucose was measured in triplicate by the glucose oxidase method (glucose analyzer, Beckman Instruments, Palo Alto, CA, USA), with a CV of 1.2 %. Leptin and adiponectin were measured by radioimmunoassay using commercially available kits (Leptin: Mediadiagnost; Adiponectin: DRG Diagnostic); insulin was measured with a chemiluminescent immunoassay (Siemens Immulite 2000). The assay sensitivity was 1 ng/mL, and inter- and intra-assay CVs were less than 10, 5, and 6 % for leptin, adiponectin, and insulin, respectively. Thyroid-stimulating hormone (TSH), free thyroxine (T4), and free triiodothyronine (T3) were measured by automated chemiluminescence methods (ACS 180 SE; Bayer, Milan, Italy). Plasma testosterone was determined using Testosterone II (Roche Diagnostics, Indianapolis, IN, USA) performed on Modular Analytics E 170 analyzer with electrochemiluminescent detection.

### Strength tests

One repetition maximum (1-RM) for the leg press and the bench press exercises was measured on separate days. Subjects executed a specific warm-up for each 1-RM test by performing 5 repetitions with a weight they could normally lift 10 times. Using procedures described elsewhere [45], the weight was gradually increased until failure occurred in both of the exercises tested. The greatest load lifted was considered the 1-RM. Previously published ICCs for test–retest reliability for leg press and bench press 1-RM testing was 0.997 and 0.997, respectively, in men, with a coefficient of variation of 0.235 for LP and 0.290 for BP [46]. 1-RM was also assessed at baseline and after 4 and 8 weeks for all training exercises so that the necessary adjustments for possible strength increases could be made, thus ensuring that subjects continued to train at a relative intensity of 85–90 % of their 1-RM.

### Statistical analysis

Results are presented as mean ± standard deviation. The sample size was obtained assuming an interaction of a Root Mean Square Standardized Effect (RMSSE) of 0.25 with a fixed power of 80 % and an alpha risk of 5 % for the main variable. Through the Shapiro–Wilk’s W test, we assessed the normality. An independent samples t test was used to test baseline differences between groups. The two-way repeated-measures ordinary ANOVA was performed (using time as the within-subject factor and diet as the between-subject factor) in order to assess differences between groups over the course of the study. Moreover we adopted a mixed model ANOVA with the fixed variable fat mass expressed in kg as covariate vs Time * Diet as random variables. All differences were considered significant at P < 0.05. Post-hoc analyses were performed using the Bonferroni test. In order to reduce the influence of within group variability a univariate test of significance (ANCOVA) was performed. We fixed as depended variable the Δ pre-post for each group and the baseline values of the outcomes were adopted as covariate; IF vs ND were assumed as categorical predictors.

The analysis was performed through STATISTICA software (Vers. 8.0 for Windows, Tulsa, USA) and Prism 5 GraphPad software (Abacus Concepts GraphPad Software, San Diego, USA).

## Results

After 8 weeks, a significant decrease in fat mass was observed in the TRF group (−16.4 vs −2.8 % in ND group), while fat-free mass was maintained in both groups (+0.86 vs +0.64 %). The same trend was observed for arm and thigh muscle cross-sectional area. Leg press maximal strength increased significantly, but no difference was present between treatments. Total testosterone and IGF-1 decreased significantly in TRF after 8 weeks while no significant differences were detected in ND. Blood glucose and insulin levels decreased significantly only in TRF subjects and conformingly a significant improvement of HOMA-IR was detected. In the TRF group, adiponectin increased, leptin decreased (but this was not significant when normalized for fat mass), and T3 decreased significantly compared to ND, without any significant changes in TSH. No significant changes were detectable for lipids (total cholesterol, HDL-c and LDL-c), except for a decrease of TG in TRF group. TNF-α and IL-1β were lower in TRF at the conclusion of the study as compared to ND. A significant decrease of respiratory ratio in TRF group was recorded (Tables 3, 4).

**Table 3:** Major results of experiment with statistics adopted highlighted in italics text

	IF pre	IF post	ΔIF *t test*	ND pre	ND post	ΔND *t test*	*2 Way ANOVA Time* * *Diet*
FFM (kg)	73.08 ± 3.88	73.72 ± 4.27	n.s.	73.93 ± 3.9	74.41 ± 3.59	n.s.	n.s.
FM (kg)	10.90 ± 3.51	9.28 ± 2.47	0.0005	11.36 ± 4.5	11.05 ± 4.274	n.s.	0.0448
Arm muscle CSA (cm^2^)	48.52 ± 3.80	49.37 ± 3.66	n.s.	48.93 ± 4.05	50.17 ± 6.27	n.s.	n.s.
Thigh CSA (cm^2^)	148 ± 34.87	153.77 ± 36.83	n.s.	150.26 ± 22.21	157.35 ± 32.56	n.s.	n.s.
Bench press 1-RM (kg)	107.08 ± 18.01	110.36 ± 16.53	n.s.	109.82 ± 14.72	110.57 ± 15.11	n.s.	n.s.
Leg press 1-RM (kg)	282.8 ± 30.11	290.00 ± 27.77	n.s.	298.56 ± 25.76	309 ± 68.94	n.s.	n.s.
Adiponectin (μg/mL)	11.8 ± 4.3	13.9 ± 3.7	0.0001	10.8 ± 5.5	10.9 ± 4.3	n.s.	0.0000
Leptin (ng/mL)	2.1 ± 0.6	1.8 ± 0.4	0.0002	2.4 ± 0.5	2.3 ± 0.4	n.s.	0.0001
Leptin (ng/mL/kg bw)	0.21 ± 0.07	0.2 ± 0.06	n.s.	0.24 ± 0.11	0.24 ± 0.11	n.s.	n.s.
IL-6 (ng/L)	1.33 ± 0.23	1.08 ± 0.22	0.0035	1.24 ± 0.38	1.19 ± 0.33	n.s.	n.s.
TNF-α (ng/L)	5.58 ± 0.92	5.13 ± 0.8	0.0001	5.69 ± 0.77	5.86 ± 0.72	n.s.	n.s.
IL-1β (ng/L)	0.93 ± 0.19	0.81 ± 0.07	0.0042	0.92 ± 0.12	0.94 ± 0.12	n.s.	0.0235
Testosterone total (nmol/L)	21.26 ± 6.51	16.86 ± 4.25	0.0001	18.60 ± 5.68	18.85 ± 4.57	n.s.	0.0476
IGF-1 (ng/mL)	216.94 ± 49.55	188.90 ± 31.48	0.0109	215.59 ± 56.25	218.41 ± 42,24	n.s.	0.0397
Insulin (mU/mL)	2.78 ± 0.6	1.77 ± 0.9	0.0303	2.56 ± 0.5	2.22 ± 0.4	n.s.	n.s.
TSH (mUI/L)	1.28 ± 0.6	1.27 ± 0.7	n.s.	1.30 ± 0.8	1.31 ± 0.6	n.s.	n.s.
T3 (ng/dL)	83.21 ± 17.23	74.32 ± 26.66	0.0001	81.12 ± 20.00	82.35 ± 25.55	n.s.	n.s.
Glucose (mg/dL)	96.64 ± 5.1	85.92 ± 7.13	0.0011	95.21 ± 47.77	96.02 ± 65.32	n.s.	n.s.
Total cholesterol (mg/dL)	193.45 ± 6.6	191.37 ± 11.2	n.s.	196.33 ± 9.93	197.12 ± 15.66	n.s.	n.s.
Cortisol (ng/mL)	174.25 ± 56.78	186.05 ± 68.5	n.s.	191.24 ± 70.34	185.78 ± 65.89	n.s.	n.s.
HDL-c (mg/dL)	54.11 ± 5.89	58.06 ± 6.11	0.0142	53.33 ± 9.67	54.12 ± 9.9	n.s.	n.s.
LDL-c (mg/dL)	114.58 ± 11.33	110.26 ± 12.27	n.s.	115.58 ± 9.9	116.08 ± 11.56	n.s.	n.s.
TG (mg/dL)	123.78 ± 15.12	115.23 ± 11.77	0.0052	137.10 ± 16.98	134.58 ± 15.66	n.s	0.0201
REE (kcal/day)	1880 ± 94.15	1891 ± 100.56	n.s.	1901 ± 88.76	1895 ± 93.56	n.s.	n.s.
RR	0.83 ± 0.02	0.81 ± 0.01	0.0421	0.83 ± 0.03	0.83 ± 0.02	n.s.	n.s.
							*Mixed model ANOVA with FM as covariate*
Adiponectin (μg/mL)	11.8 ± 4.3	13.9 ± 3.7		10.8 ± 5.5	10.9 ± 4.3		0.0000
Leptin (ng/mL)	2.1 ± 0.6	1.8 ± 0.4		2.4 ± 0.5	2.3 ± 0.4		0.0002
Leptin (ng/mL/kg bw)	0.21 ± 0.07	0.2 ± 0.06		0.24 ± 0.11	0.24 ± 0.11		0.0135
IL-1β (ng/L)	0.93 ± 0.19	0.81 ± 0.07		0.92 ± 0.12	0.94 ± 0.12		0.0224

Results are presented as mean ± SD

*n.s.* not statistically significantly different (p > 0.05)

**Table 4:** Univariate tests of significance (ANCOVA)

Univariate tests of significance (ANCOVA)				
Observations	Dependent variables (pre–post Δ)	Categorical predictors (ΔIF vs ΔND)	Covariates (baseline values)	P values
Body weight	−0.40 ± 1.76	−0.97 ± 1.58 vs 0.16 ± 1.78	84.63 ± 6.17	0.0354
FM (kg)	−0.96 ± 1.72	−1.61 ± 1.53 vs −0.30 ± 1.70	11.12 ± 3.98	0.0070
Adiponectin (μg/mL)	−0.45 ± 3.07	2.04 ± 1.52 vs −2.94 ± 1.97	12.83 ± 1.98	0.0000
Leptin (ng/mL)	0.09 ± 0.67	−0.36 ± 0.31 vs 0.54 ± 0.64	1.97 ± 0.52	0.0000
IL-6 (ng/L)	−0.15 ± 0.27	−0.25 ± 0.30 vs −0.04 ± 0.20	1.28 ± 0.31	0.0378
TNF-α (ng/L)	−0.14 ± 0.47	−0.45 ± 0.37 vs 0.17 ± 0.34	5.63 ± 0.83	0.0000
IL-1β (ng/L)	−0.05 ± 0.14	−0.12 ± 0.15 vs 0.02 ± 0.09	0.92 ± 0.15	0.0000
Testosterone total (nmol/L)	−2.07 ± 3.60	−4.40 ± 3.02 vs 0.25 ± 2.43	19.93 ± 6.00	0.0000
IGF-1 (ng/mL)	−12.38 ± 33.04	−28.00 ± 40.11 vs 3.23 ± 11.13	216.32 ± 38.84	0.0003

The Δ pre–post for each depended variable group were considered and the baseline values of the outcomes were adopted as covariate; TRF vs ND were assumed as categorical predictors

## Discussion

Fasting is a relatively well-studied metabolic state in sports and physical exercise due to studies of the “Ramadan” period observed by Muslim athletes [12, 14]. However, only a single study has reported its effect during a resistance training program aimed at achieving skeletal muscle growth [30]. Our data demonstrate that during a RT program, TRF was capable of maintaining muscle mass, reducing body fat, and reducing inflammation markers. However, it also reduced anabolic hormones such testosterone and IGF-1.

A key point of the TRF approach utilized in the present study is that total daily calorie intake remained the same while the frequency of meals (i.e. time between meals) was altered. This is dissimilar to many other IF regimens. There are a number of different IF protocols, most of which have the goal of reducing total energy intake. Additionally, unlike ADF and some other forms of IF, the regimen utilized in the present study employed the same schedule each day, consisting of 16 h fasting and 8 h feeding.

Although IF has received a great amount of attention in recent years, the majority of studies have investigated the effects of IF in overweight, obese or dyslipidemic subjects [19–21, 47–50]. However, little is known about the effects of such nutritional regimens in athletes, and more specifically, in body builders or resistance-trained individuals. The present study provides the first in-depth investigation of IF in this population of athletes. With the exception of reduced triglycerides, our results do not confirm previous research suggesting a positive effect of IF on blood lipid profiles [17–19, 47, 49, 51, 52], however, it has to be taken into account that our subjects were normolipemic athletes. The magnitude of reduction in triglycerides was also smaller than is typically seen in individuals who have elevated concentrations prior to IF.

As reported, a decrease of fat mass in individuals performing IF was observed. Considering that the total amount of kilocalories and the nutrient distribution were not significantly different between the two groups (Table 2), the mechanism of greater fat loss in IF group cannot simply be explained by changes in the quantity or quality of diet, but rather by the different temporal meal distribution. Many biological mechanisms have been advocated to explain these effects. One is the increase of adiponectin that interacts with adenosine 5′-monophosphate-activated protein kinase (AMPK) and stimulates Peroxisome proliferator-activated receptor gamma coactivator 1-alpha (PGC-1α) protein expression and mitochondrial biogenesis. Moreover, adiponectin acts in the brain to increase energy expenditure and cause weight loss [53]. It is notable that in the present study, the differences in adiponectin between groups remained even when normalized relative to body fat mass, whereas the significant decrease of leptin (that might be considered a unfavorable factor for fat loss) was no longer significant when normalized for fat mass. Other hypothesis is an enhanced thermogenic response to epinephrine [54] or an increase in REE [55] after brief periods of fasting, but our preliminary data didn’t support this point.

Interestingly, although reductions in the anabolic hormones testosterone and IGF-1 were observed, this did not correspond to any deleterious body composition changes or compromises of muscular strength over the duration of the study. It has been previously reported that men performing caloric restriction have lower testosterone than those consuming non-restricted Western diets [56], however, the present experiment did not restrict calories in the IF group. In animal models, IF influences the hypothalamo-hypophysial-gonadal axis and testosterone concentration probably through a decrease in leptin-mediated effects [57], but it must be considered that mice on a an every-other-day feeding regimen consume about 30–40 % less calories over time compared to free feeding animals and that in our study, no differences in leptin concentration were seen when normalized for fat mass. Also, the reduction of IGF-1 in the TRF group deserves some discussion. A previous study by Bohulel et al. [11] reported no changes in the GH/IGF-1 during Ramadan intermittent fasting. Even though it is plausible that IF mimics caloric restriction through common pathways (e.g. AMPK/ACC) (adenosine 5′-monophosphate-activated protein kinase/acetyl-CoA-carboxylase) [58], recent data on humans showed no influences of caloric restriction on IGF-1 [59, 60]. It is possible that the increase of adiponectin and the decrease of leptin could influence the IGF-1 concentration, even though it is unclear to what extent changes in adipokines impact circulating IGF-1 levels following weight loss [59].

Previous studies have reported mixed results concerning the ability to maintain lean body mass during IF, but the vast majority of these studies imposed calorie restriction and did not utilize exercise interventions [22]. In our study, the nutrient timing related to training session was different between the two groups, and this could affect the anabolic response of the subjects [61] even though these effects are still unclear [62]. However, we did not find any significant differences between groups in fat-free mass, indicating that the influence of nutrient timing may be negligible when the overall content of the diet is similar.

There is an increasing amount of data suggesting that IF could potentially be a feasible nutritional scheme to combat certain diseases. In the present study, both blood glucose and insulin concentrations decreased in the IF group. The potential of IF to modulate blood glucose and insulin concentrations has previously been discussed, but primarily in the context of overweight and obese individuals [3]. The concurrent increase in adiponectin and decrease in insulin may be related to modulation of insulin sensitivity, as adiponectin concentrations have been positively correlated with insulin sensitivity [21, 50, 63, 64]. Moreover, related to the well-known anti-inflammatory effect of adiponectin, it is possible that the reduction of inflammatory markers is related to the improvement of insulin sensitivity. Inflammation plays an pivotal role in insulin resistance development through different cytokines that influence numerous molecular pathways. For example, insulin resistance could be triggered by TNF-*α* via JNK and IKK*β*/NF-*κ*B (jun amino-terminal kinase/inhibitor of NF-*κβ* kinase) pathways, which may increase serine/threonine phosphorylation of insulin receptor substrate 1. Moreover IL-6 could decrease insulin sensitivity in skeletal muscle by inducing toll-like receptor-4 (TLR-4) gene expression through STAT3 (activator of transcription 3) activation. This relationship is potentially bidirectional as the activation of IKK*β*/NF-*κ*B signalling could, in turn, stimulate the production of TNF-*α* [65]. Modulation of some of these inflammatory markers by IF was seen in the present study: TNF-α and IL-1β were lower in the TRF group than ND at the conclusion of the study, while IL-6 appeared to decrease in the TRF group, but was not significantly different from ND. Previous information on the impact of IF on inflammatory markers is limited, but a previous investigation by Halberg et al. [66] reported no changes in TNF-α or IL-6 after two weeks of modified IF in a small sample of healthy young men.

Although a reduction in T3 was observed in the IF group, no changes in TSH or resting energy expenditure were observed. The observed reduction in RR in the TRF group indicates a very small shift towards reliance on fatty acids for fuel at rest, although a significant statistical interaction for RR was not present. Fasting RR has been previously reported to be a predictor of substantial future weight gain in non-obese men, with individuals who have higher fasting RR being more likely to gain weight [67]. Interestingly, it was reported by Seidell et al. [67] that although RR was related to future weight gain, RMR was not. It should be noted that individuals with the highest risk of future weight gain had fasting RR > 0.85 (as compared to individuals who had RR < 0.76). In the present study, the RR at the end of the study in both the TRF group and ND group do not directly fall into either of these categories (RR = 0.81 and 0.83, respectively).

Based on the present study, a modified IF protocol (i.e. TRF) could be feasible for strength athletes without negatively affecting strength and muscle mass. Interestingly, even though androgen concentrations were lowered by TRF, there was no difference in muscle mass changes between groups (+0.64 kg in TRF vs +0.48 kg in ND). Caloric restriction in rodents has been reported to decrease testosterone and IGF-1 even though human data on long-term severe caloric restriction does not demonstrate a decrease in IGF-1 levels, but instead an increased serum insulin-like growth factor binding protein 1 (IGFBP-1) concentration [60, 68]. However, no data are available for most forms of IF. Decrease the activity of the IGF-1 axis could be a desirable target for reducing cancer risk [69], but it is also well known that the activation of the IGF-1/AKT/mTOR (insulin-like growth factor-1/protein kinase B/mammalian target of rapamycin) pathway is one of the keys for muscular growth. In addition to altering IGF-1, fasting can promote autophagy [28], which is important for optimal muscle health [70]. Additionally, there is a possibility that the different eating patterns of the groups in the present study impacted the relative contributions of different hypertrophic pathways in each group.

Some limitations of the present study should be taken into account. One is the different timing of meals in relationship to the training sessions that could have affected the subjects’ responses. On this point, there is not a consensus among researchers. The beneficial effects of pre-exercise essential amino acid-carbohydrate supplement have been suggested [61], but the same group found that ingesting 20 g of whey protein either before or 1 h after 10 sets of leg extension resulted in similar rates of AA uptake [62]. Additionally, other studies have reported no benefit with pre-exercise AA feeding [71, 72]. Another limitation of the present study is that the energy and macronutrient composition of the diet was based on interview, and this approach has known weaknesses. Because of the limitations of this method, it is possible that differences in energy or nutrient intake between groups could have existed and played a role in the observed outcomes.

## Conclusions

In conclusion, our results suggest that the modified IF employed in this study: TRF with 16 h of fasting and 8 h of feeding, could be beneficial in resistance trained individuals to improve health-related biomarkers, decrease fat mass, and at least maintain muscle mass. This kind of regimen could be adopted by athletes during maintenance phases of training in which the goal is to maintain muscle mass while reducing fat mass. Additional studies are needed to confirm our results and to investigate the long-term effects of IF and periods after IF cessation.

## Abbreviations

IF: intermittent fasting
TRF: time-restricted feeding
ND: normal diet
ADF: alternate day fasting
IL-6: interleukin-6
TNF-α: tumor necrosis factor-α
IL-1β: interleukin-1β
IGF-1: insulin-like growth factor-1
HDL-C: high-density lipoprotein cholesterol
LDL-C: low-density lipoprotein cholesterol
TG: triglycerides
TSH: thyroid-stimulating hormone
T4: free thyroxine
T3: free triiodothyronine
1-RM: one repetition maximum
REE: resting energy expenditure
RR: respiratory ratio
ACC: acetyl-CoA-carboxylase
AMPK: adenosine 5′-monophosphate-activated protein kinase
PGC-1α: Peroxisome proliferator-activated receptor gamma coactivator 1-alpha
HOMA-IR: homeostasis model assessment–insulin-resistance
mTOR: mammalian target of rapamycin
AKT: protein kinase B
IGFBP-1: insulin-like growth factor binding protein 1
JNK: jun amino-terminal kinase
IKK*β*/NF-*κ*B: inhibitor of NF-*κβ* kinase
STAT3: activator of transcription 3
TLR-4: toll-like receptor-4
